# Biogenesis and downstream effects of 3′,5′ and 2′,3′ cAMP isomers in plants

**DOI:** 10.1126/sciadv.aea7828

**Published:** 2026-05-08

**Authors:** Mingyue Li, Monika Chodasiewicz, Malavika Muraleedharan, Israel M. Lopez, Michal Gorka, Olga Kerber, Saqer S. Alotaibi, Andrew D.L. Nelson, Rene Lenobel, Jaroslava Friedecká, Aleksandra Skirycz, Jiří Friml

**Affiliations:** ^1^ Institute of Science and Technology Austria (ISTA), 3400 Klosterneuburg (Austria).; ^2^ Biological and Environmental Science & Engineering Division (BESE), King Abdullah University of Science and Technology (KAUST), Thuwal, Saudi Arabia.; ^3^ Laboratory of Growth Regulators, Faculty of Science, Palacký University and Institute of Experimental Botany of the Czech Academy of Sciences, Šlechtitelů 27, CZ-783 71 Olomouc, Czech Republic.; ^4^ Michigan State University, East Lansing, MI 48824, USA.; ^5^ Max-Planck-Institute of Molecular Plant Physiology, 14476 Potsdam-Golm, Germany.; ^6^ Department of Biotechnology, College of Science, Taif University, P.O. Box 11099, Taif 21944, Saudi Arabia.; ^7^ The Boyce Thompson Institute, Cornell University, Ithaca, NY 14853, USA.

## Abstract

Cyclic adenosine monophosphate (cAMP) is a fundamental second messenger involved in diverse signaling pathways across both animals and plants. While the role of 3′,5′-cAMP has been extensively characterized, the biological significance of its structural isomer, 2′,3′-cAMP, remains largely unexplored, particularly in plants. Here, we show that 2′,3′-cAMP and 3′,5′-cAMP represent parallel signaling systems in *Arabidopsis thaliana*, with different enzymatic origins and largely distinct downstream effects. In vitro enzymatic assays show that plant adenylate cyclases (ACs), including AFB5 and HpAC1, produce specifically 3′,5′-cAMP from ATP, whereas the TIR domain of protein L7 also catalyzes the formation of 2′,3′-cAMP from RNA. Comprehensive multiomics analyses reveal that two isomers elicit distinct yet partially overlapping metabolic, proteomic, and transcriptional response: 2′,3′-cAMP activates broad, stress-adaptive gene expression reprogramming, while 3′,5′-cAMP fine-tunes responses related to nutrient status and cellular homeostasis. Our findings establish the existence of dual cAMP signaling systems in plants, each with specialized functions and provide insights into the complex regulatory networks governing plant physiology.

## Teaser

2′,3′-cAMP and 3′,5′-cAMP act as distinct but partially overlapping regulators of plant development and physiology.

## INTRODUCTION

Cyclic nucleotides (cNMPs), notably cyclic adenosine monophosphate (cAMP), have been well-established as pivotal second messengers in animal systems, orchestrating processes such as neurotransmission, hormone signaling, and metabolic regulation (*1*). In contrast, the roles of cNMPs in plants have remained less defined, with early studies suggesting the presence of cAMP in plant tissues but lacking clarity on its biological significance due to generally lower endogenous levels and the absence of well-characterized cAMP-dependent pathway (*2*, *3*). Recent advancements have revitalized interest in plant cAMP signaling, particularly with the research focusing on identification of adenylate cyclases (ACs) and guanylate cyclases (GCs) (*4*–*6*). Unlike their animal counterparts, characterized plant ACs and GCs often display lower catalytic activity, leading to ongoing debates regarding their in vivo significance (*7*). Notably, the identification of cAMP isomer production by key plant proteins, such as the Toll/interleukin-1 receptor 1 (TIR1)/Auxin Signaling F-box (AFB) receptors for the plant hormone auxin (*8*) and TIR-domain containing L7 protein (*9*), has provided further insights into cAMP’s involvement in hormone signaling and stress adaption, respectively.

A critical aspect of cAMP signaling that has garnered attention is the existence of distinct isomers, 3′,5′-cAMP and 2′,3′-cAMP, and their respective roles in plant biology. In mammals, 2′,3′-cAMP has been associated with RNA degradation pathways and stress responses (*10*), whereas 3′,5′-cAMP functions in classical signaling cascades downstream of G protein–coupled receptors (*11*). Initial observations suggest that plants may similarly use these isomers in distinct ways. For example, 3′,5′-cAMP, but not 2′,3′-cAMP, showed to influence actin cytoskeleton (*12*). In contrast, 2′,3′-cAMP but not 3′,5′-cAMP was directly linked into stress signaling by its ability to bind to RNA binding protein 47 b (RBP47b) and induce stress granule (SG) formation (*13*). In line with the discovery of the link between 2′,3′-cAMP and stress responses (*14*), researchers identified TIR domains of plant immune receptors as nucleic acid hydrolases/ 2′,3′-cNMP synthetases that mediate cell death in response to pathogen attack (*9*). Here, 2′,3′-cAMP was identified as being required for TIR-mediated cell death in plants highlighting the importance of this signaling molecule.

On the other hand, the predominantly nuclear auxin receptors TIR1/AFB generate the canonical 3′,5′-cAMP isomer (*8*), which serves as an indispensable second messenger for activating downstream transcriptional reprogramming (*15*). This finding revises the well-established canonical mechanism of nuclear auxin signaling, yet major gaps still remain, not least the identity of 3′,5′-cAMP target(s) and the mechanisms governing 3′,5′-cAMP inactivation in plants (*16*).

Despite these initial insights, many fundamental questions remain open regarding the biogenesis, metabolism, regulatory mechanisms, and specific functions of cAMP isomers in plants. While the production of 3′,5′-cAMP is primarily attributed to enzymes with AC activity, the enzymatic source of 2′,3′-cAMP is less well-defined. In addition, the spatial and temporal dynamics of these molecules within plant cells require further elucidation. Understanding these aspects is crucial for determining how plants integrate cAMP signaling into broader physiological and developmental frameworks.

This study aims to characterize the origin, metabolism, and functional effects of 3′,5′-cAMP and 2′,3′-cAMP in plants using a combination of analytical, metabolomic, transcriptomic, and proteomic approaches. By examining the distinct and overlapping roles of these isomers, we seek to provide insights into CNP signaling in plant systems, potentially uncovering regulatory pathways with implications for growth, development, and stress adaptation.

## RESULTS

### Analytical detection of 2′,3′-cAMP and 3′,5′-cAMP isomers in plant tissues

**Fig. 1.**
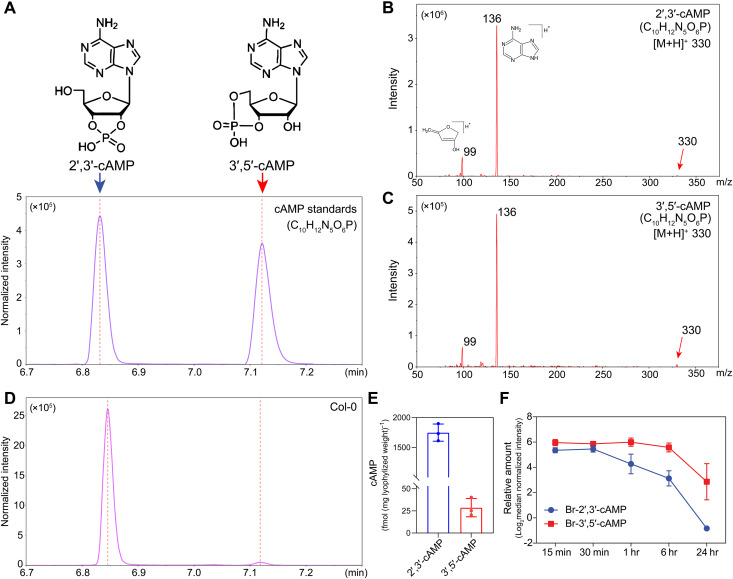
Detection of 2′,3′-cAMP and 3′,5′-cAMP in planta by HPLC-MS. (**A**) Separation of 2′,3′-cAMP and 3′,5′-cAMP standards analyzed by LC-MS in multiple reaction monitoring (MRM) mode. Molecular structure of 2’,3’-cAMP and 3′,5′-cAMP are shown on top, with retention time peaks corresponding to each compound indicated. (**B** and **C**) Comparison of the triple quadrupole MS spectra of standard 2′,3′-cAMP (B) and 3′,5′-cAMP (C). Theoretical [M + H]^+^ for both isomers: mass-to-charge (*m*/*z*) 330; major fragment ions: *m*/*z* 99 and 136. Data are shown as representative chromatograms from three independent experiments. (**D**) Detection of endogenous 2′,3′-cAMP and 3′,5′-cAMP in *Arabidopsis* by LC-MS. (**E**) Quantification of detected 2′,3′-cAMP and 3′,5′-cAMP isomers in (D). *n* = 3 biological replicates. (**F**) Dynamic changes in Br-2′,3′-cAMP and Br-3′,5′-cAMP levels *in planta* following treatment. Data represent mean log_2_ median-normalized intensity ± SD (*n* = 4).

To investigate the presence and dynamics of cAMP isomers in plants, we optimized and performed high-performance liquid chromatography–mass spectrometry (HPLC-MS) analysis on *Arabidopsis* seedlings. The chromatographic separation of authentic 2′,3′-cAMP and 3′,5′-cAMP standards was established (Fig. 1A), enabling reliable identification and quantification of these isomers (Fig. 1, B and C). Quantitative analysis revealed that both 2′,3′-cAMP and 3′,5′-cAMP are endogenously present in *Arabidopsis* tissues (Fig. 1, D and E). Basal 2′,3′-cAMP level was significantly higher than 3′,5′-cAMP, reaching lyophilized weight of 1748.7 fmol/mg compared with 28.7 fmol/mg for 3′,5′-cAMP (Fig. 1E). These values are consistent with previous plant measurement: 2′,3′-cAMP has been reported at 255 to 1664 fmol/mg tissue in *Arabidopsis* leaves (*17*), and the 3′,5′-cAMP levels are of the same order of magnitude across plant species, with ~17 fmol/mg in *Brassica napus* seedling (*18*) and a fresh weight of <12 fmol/mg in *Lilium longiflorum* pistils (*19*). In contrast, 3′,5′-cAMP level in mammalian spans a broader range, from ~36 fmol/mg in rabbit pancreas to 488 fmol/mg in rat heart (*20*), whereas elevated 2′,3′-cAMP level is considered potentially toxic and therefore under tightly controlled, presenting ~10 fmol/mg in rat brain (*20*). Collectively, these comparisons imply substantial differences between plant and mammalian systems in the regulation and metabolism of cAMP isomers.

To assess uptake and in planta metabolic dynamics of these isomers, Col-0 seedlings were treated with 1 μM Br-2′,3′-cAMP or Br-3′,5′-cAMP and sampled after 15 min, 30 min, 1 hour, 6 hours, and 24 hours. Both isomers showed time-dependent accumulation and clearance patterns, but with distinct kinetics. Br-2′,3′-cAMP levels peaked at 30 min posttreatment, declined sharply at 1 hour, and continued to decrease over time, with only trace amounts detectable at 24 hours (Fig. 1F). This rapid turnover suggests a highly dynamic metabolic regulation. In contrast, Br-3′,5′-cAMP levels remained relatively stable from 15 min to 6 hours posttreatment, with a noticeable decrease only at 24 hours. Substantial amounts of Br-3′,5′-cAMP remained detectable at 24 hours, indicating greater metabolic stability compared to its 2′,3′-isomer (Fig. 1F).

These observations show that both, 2′,3′ and 3′,5′ cAMP isomers can be reliably detected in plants with the 2′,3′-cAMP being more abundant and also more strictly metabolically controlled.

### Distinct substrate and product specificities of enzymes producing the cAMP isomers

**Fig. 2.**
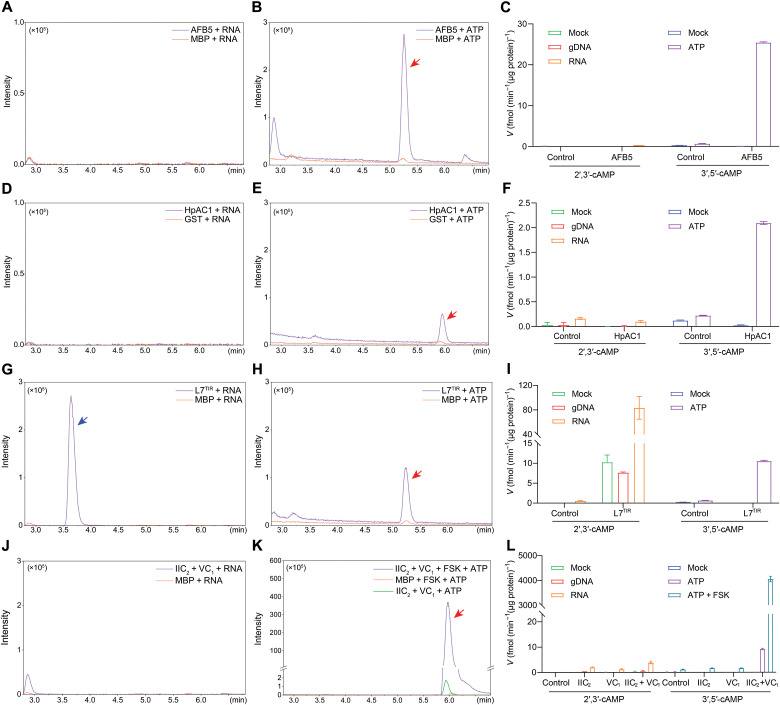
Distinct enzymatic origins of 2′,3′-cAMP and 3′,5′-cAMP in plants and mammals. Quantification of 2′,3′-cAMP production using genomic DNA (gDNA) or RNA as substrates and 3′,5′-cAMP production using ATP as a substrate by various recombinant enzymes: (**A** to **C**) 5 μg of AFB5 from *Arabidopsis*. (**D** to **F**) 5 μg of *Hippeastrum* AC 1 (HpAC1). (**G** to **I**) 5 μg of TIR domain of the flax TNL protein L7 (L7^TIR^) from *Linum usitatissimum*. (**J** to **L**) Reconstituted mammalian AC composed of 2.5 μg of C1a domain from type V adenylyl cyclase (VC_1_) and 2.5 μg of C2 domain from type II adenylyl cyclase (IIC_2_), with or without 10 μM forskolin (FSK) stimulation. Proteins are incubated with gDNA or total RNA at 25°C for 16 hours or incubated with 1 mM ATP at 30°C for 25 min. Reaction products were analyzed by LC-MS in MRM mode. Representative MRM chromatograms are shown for 2′,3′-cAMP [(A), (D), (G), and (J)] and 3′,5′-cAMP [(B), (E), (H), and (K)]. Retention time peaks corresponding to 2′,3’-cAMP and 3′,5′-cAMP are indicated by blue and red arrows, respectively. Quantification of the reaction products is shown in (C), (F), (I), and (L). Data represent means ± SD (*n* = 3 biological replicates).

An important question in CNP biology is whether the 2′,3′-cAMP and 3′,5′-cAMP isomers can be produced by the same enzyme, or whether they are synthesized by distinct enzymatic mechanisms. To address this, we performed in vitro enzymatic assays using purified recombinant plant enzymes with previously suggested activities as AC (to produce 3′,5′-cAMP) or CNP synthase (to produce 2′,3′-cAMP) (Fig. 2 and figs. S1 and S2). Each enzyme was incubated either with nucleic acid substrates (genomic DNA or RNA) for 16 hours or with adenosine 5′-triphosphate (ATP) for 25 min, and the production rates of the respective cAMP isomers were quantified.

Our results reveal clear substrate and product specificity across the enzymes tested. The plant-derived ACs, AFB5 (*8*) and *Hippeastrum* AC 1 (HpAC1) (*21*), primarily generated 3′,5′-cAMP from ATP (Fig. 2, B and E), whereas incubation with double-stranded DNA (dsDNA)/RNA yielded only trace amounts of 2′,3′-cAMP (Fig. 2, A and D). AFB5 exhibited higher AC activity than HpAC1 (Fig. 2, C and F). Consistently, the Michaelis-Menten kinetics analysis of AFB5 indicated a maximum velocity (*V*_max_) of 46.06 fmol min^−1^ μg^−1^ and a Michaelis constant (*K*_m_) of 0.1833 mM (fig. S2A). To further confirm its activity, we introduced two mutations in conserved residues within the AC motif (E597A and D611A), both of which remarkably reduced AFB5 AC activity (fig. S2B), in agreement with previous study (*8*).

A similar pattern was observed in the reconstituted mammalian AC composed of the C1a domain from type V adenylyl cyclase (VC_1_) and the C2 domain from type II adenylyl cyclase (IIC_2_), which predominantly catalyzed the formation of 3′,5′-cAMP (Fig. 2, J to L). The expression of either VC_1_ or IIC_2_ alone did not yield detectable levels of either cAMP isomer. In contrast, the reconstitution of VC_1_ with IIC_2_ resulted in 3′,5′-cAMP production with a velocity of 9.17 fmol min^−1^ μg^−1^ (Fig. 2L). Notably, the addition of forskolin (FSK), a known activator of mammalian ACs (*22*), significantly enhanced 3′,5′-cAMP production to 4049.6 fmol min^−1^ μg^−1^ (Fig. 2L), corresponding to an ~88-fold higher activity than that of AFB5.

By contrast, the TIR domain of the flax TNL protein L7 (L7^TIR^), previously identified as a CNP synthetase involved in TIR-mediated cell death, robustly produced 2′,3′-cAMP from nucleic acid substrates, with RNA being a more efficient substrate than DNA (Fig. 2, G and I), consistent with previous reports (*9*). Substituting cysteine-132 at the nucleotide pocket (C132A) significantly abolished the 2′,3′-cAMP synthesis (fig. S2D), supporting the proposed synthesis activity (*9*). Unexpectedly, L7^TIR^ also exhibited the ability to catalyze 3′,5′-cAMP production from ATP (Fig. 2, H and I). The kinetics analysis of L7^TIR^ AC activity identified a *V*_max_ of 14.70 fmol min^−1^ μg^−1^ and a *K*_m_ of 0.2032 mM (fig. S2C).

Together, these findings indicate that 2′,3′-cAMP and 3′,5′-cAMP isomers are preferentially produced by different enzymes with distinct substrate specificities. However, some enzymes such as L7^TIR^ may display dual activity, suggesting a potential evolutionary or functional flexibility. The substrate-dependent specificity observed across multiple ACs reinforces the notion that these two cAMP isomers arise from biochemically distinct mechanisms.

### Global effects of 3′,5′-cAMP and 2′,3′-cAMP isomers on metabolome

To investigate whether 2′,3′-cAMP and 3′,5′-cAMP elicit distinct cellular responses, *Arabidopsis* seedlings were treated with membrane-permeable analogs, Br-2′,3′-cAMP (*14*) and Br-3′,5′-cAMP. Previous studies suggested that Br-2′,3′-cAMP can mimic stress responses in plants (*14*). Liquid-grown seedlings were treated with 1 μM of either compound or mock solution as a control. Samples were collected at 15 min, 30 min, 1 hour, 6 hours, and 24 hours posttreatment. Global responses at metabolome, proteome, and transcriptome level were carefully analyzed to uncover both differential and overlapping effects.

**Fig. 3.**
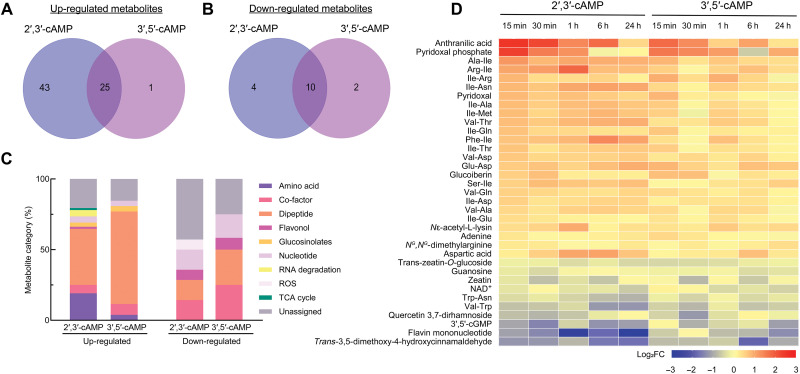
Comparative metabolomics after 2′,3′-cAMP and 3′,5′-cAMP treatment. (**A**) Venn diagram showing an overlap of significantly up-regulated metabolites in response to 1 μM Br-2′,3′-cAMP or Br-3′,5′-cAMP treatment. (**B**) Venn diagram showing the overlap of significantly down-regulated metabolites following the same treatments. (**C**) Classification of metabolites significantly up- or down-regulated based on two-way ANOVA with FDR-corrected *P* ≤ 0.05. (**D**) Heatmap depicting shared significant changes in metabolite levels. Data are presented as log_2_ fold changes (two-way ANOVA, FDR-corrected *P* ≤ 0.05).

Comparative metabolomic profiling identified 85 primary and specialized metabolites, of which 69 were significantly up-regulated in response to Br-2′,3′-cAMP or Br-3′,5′-cAMP treatment [two-way analysis of variance (ANOVA), false discovery rate (FDR)–corrected *P* ≤ 0.05; Fig. 3A], and 16 metabolites were significantly down-regulated (Fig. 3B). Br-2′,3′-cAMP exerted a stronger impact, reflected by a higher number of induced metabolites compared to Br-3′,5′-cAMP (Fig. 3A). Notably, most metabolites induced by Br-3′,5′-cAMP overlapped with those induced by Br-2′,3′-cAMP (Fig. 3, A and B).

The classification of differentially regulated metabolites revealed considerable overlap between treatments, suggesting partially shared but distinct signaling pathways (Fig. 3C). Although both treatments triggered dipeptide accumulation, a greater diversity of dipeptides was observed in Br-3′,5′-cAMP–treated samples. In contrast, Br-2′,3′-cAMP treatment led to a more pronounced accumulation of amino acids. Uniquely, the accumulation of intermediates from the tricarboxylic acid cycle (TCA cycle), RNA degradation products, and flavonoids was detected only in Br-2′,3′-cAMP–treated seedlings. Among down-regulated metabolites, most belonged to similar chemical classes in both treatments; however, metabolites associated with reactive oxygen species (ROS) scavenging were significantly reduced only under Br-2′,3′-cAMP treatment (Fig. 3C).

The heatmap analysis of metabolites commonly regulated by both treatments further underscored that even among shared targets, the magnitude and, in some cases, the direction of change differed substantially between 2′,3′-cAMP and 3′,5′-cAMP (Fig. 3D). For example, many dipeptides—considered as regulatory molecules (*23*)—accumulated more rapidly and to a higher extent during early stages (15 to 30 min) of Br-2′,3′-cAMP treatment compared to Br-3′,5′-cAMP. In addition, reductions in 3′,5′-cGMP levels were more pronounced under Br-2′,3′-cAMP treatment (Fig. 3D). Together, the analysis of metabolomics profile revealed that despite some overlap of metabolic changes in responses to 2′,3′-cAMP and 3′,5′-cAMP, 2′,3′-cAMP have broader effects.

### Global effects of 3′,5′-cAMP and 2′,3′-cAMP isomers on proteome

Next, we performed comprehensive proteomic analysis of liquid-grown seedlings treated with 1 μM Br-2′,3′-cAMP or Br-3′,5′-cAMP for 15 min, 30 min, 1 hour, 6 hours, and 24 hours.

**Fig. 4.**
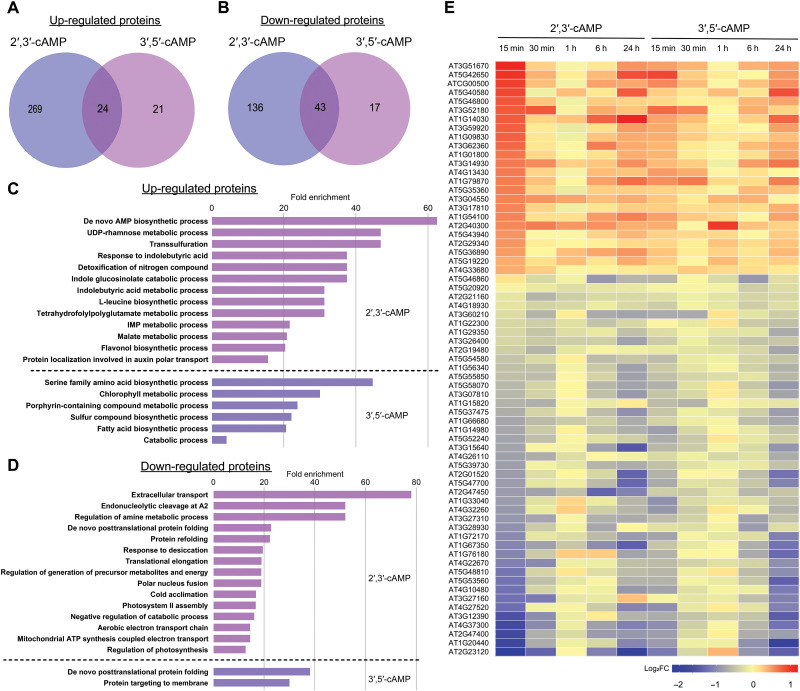
Comparative proteomics after 2′,3′-cAMP and 3′,5′-cAMP treatment. (**A**) Venn diagram showing the overlap of significantly up-regulated proteins following 1 μM Br-2′,3′-cAMP or Br-3′,5′-cAMP treatment. (**B**) Venn diagram showing the overlap of significantly down-regulated proteins in response to the same treatments. (**C**) GO term enrichment analysis of biological processes overrepresented among significantly up-regulated proteins. (**D**) GO term enrichment analysis of biological processes overrepresented among significantly down-regulated proteins. (**E**) Heatmap showing shared significant changes in protein abundance between Br-2′,3′-cAMP and Br-3′,5′-cAMP treatments. Data are presented as log_2_ fold change values (two-way ANOVA, FDR-corrected *P* ≤ 0.05, *n* = 4 biological replicates).

Similar to metabolome, 2′,3′-cAMP had stronger effect on proteome in the same time frame in comparison to 3′,5′-cAMP. Among 314 up-regulated proteins, 293 proteins were overaccumulated specifically in response to 2′,3’-cAMP treatment, with 24 proteins shared between the treatments. Only a fraction of 21 were 3′,5′-cAMP specific (Fig. 4A). Analogous response had been observed for down-regulated proteins where again most of the changes is associated with the 2′,3′-cAMP treatment (179) where only 17 are down-regulated by 3′,5′-cAMP (Fig. 4B). Subcellular localization analysis revealed an enrichment of plastid and cytosol localization among up-regulated proteins (fig. S3A), whereas an enrichment of nuclear and cytosol localization among down-regulated proteins (fig. S3B). Gene Ontology (GO) enrichment analysis of up-regulated proteins revealed distinct biological processes associated with Br-2′,3′-cAMP and Br-3′,5′-cAMP treatments. Proteins up-regulated by Br-2′,3′-cAMP were strongly enriched in processes related to nucleotide and specialized metabolism, including “de novo” AMP biosynthetic process, Uridine Diphosphate rhamnose (UDP-rhamnose) metabolic process, and transsulfuration (Fig. 4C). Additional enrichment was observed in metabolic pathways related to indole glucosinolate catabolism, detoxification of nitrogen compounds, and response to indolebutyric acid, flavonol biosynthesis, and auxin polar transport, suggesting an activation of specialized metabolic responses and stress-related pathways. By contrast, proteins up-regulated by Br-3′,5′-cAMP were predominantly enriched in broader metabolic processes, including serine family amino acid biosynthetic process, chlorophyll metabolic process, and porphyrin-containing compound metabolic process (Fig. 4C). Enrichment in sulfur compound biosynthesis and fatty acid biosynthesis pathways was also detected, indicating a reprogramming of primary metabolism.

In response to Br-2′,3′-cAMP, significantly down-regulated proteins were enriched in processes related to extracellular transport, regulation of amine metabolism, and endonucleolytic RNA cleavage involved in ribosomal RNA processing (Fig. 4D). Additional suppressed processes included de novo posttranslational protein folding, protein refolding, translational elongation, regulation of energy metabolism, cold and desiccation acclimation, and photosynthesis-related functions, suggesting broad repression of metabolic and energy-associated pathways under Br-2′,3′-cAMP treatment. By contrast, Br-3′,5′-cAMP treatment led to a much narrower set of down-regulated processes, primarily affecting de novo posttranslational protein folding and protein targeting to membranes (Fig. 4D). The heatmap of shared protein changes further illustrated that even among commonly affected proteins, the magnitude of response often differed between the two treatments (Fig. 4E), with Br-2′,3′-cAMP inducing more substantial changes in protein abundance.

Overall, these findings indicate that Br-2′,3′-cAMP and Br-3′,5′-cAMP isomers cause largely distinct effects on proteome and related biological processes with 2′,3′-cAMP, particularly affecting stress-related and specialized metabolic pathways, consistent with a more profound reprogramming of cellular energy and stress responses (*14*), while 3′,5′-cAMP primarily regulates primary metabolic processes involved in growth and maintenance.

### Global effects of 3′,5′-cAMP and 2′,3′-cAMP isomers on transcriptome

**Fig. 5.**
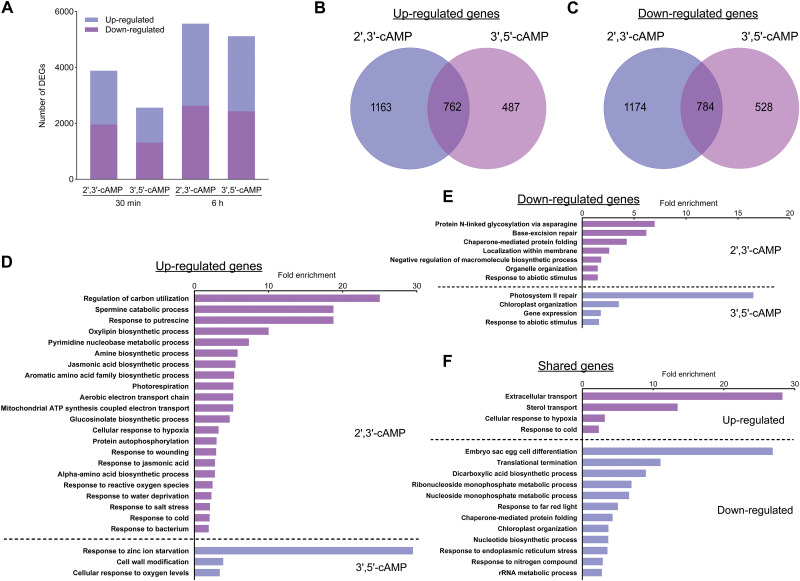
Comparative transcriptomics after 2′,3′-cAMP and 3′,5′-cAMP treatment. (**A**) Number of transcripts up-regulated or down-regulated after 30 min and 6 hours of 1 μM Br-2′,3′-cAMP or Br-3′,5′-cAMP treatment. (**B**) Venn diagram of significantly up-regulated genes following 30-min treatment. (**C**) Downregulated genes after 30-min treatment. (**D**) Overrepresentation of biological processes in the dataset of up-regulated genes. (**E**) Overrepresentation of biological processes of down-regulated genes after 30-min treatment. (**F**) Overrepresented biological processes among genes commonly regulated by both cAMP isomers, shown as fold enrichment based on the PANTHER overrepresentation test (Fisher’s exact test, FDR-corrected *P* ≤ 0.05) using *A. thaliana* as the reference background.

To examine the impact of cAMP isomers on gene expression, we performed RNA sequencing (RNA-seq) analysis at early (30 min) and late (6 hour) time points following treatment with 1 μM Br-2′,3′-cAMP or Br-3′,5′-cAMP on liquid-grown seedlings. Differentially expressed genes (DEGs) were identified as significantly up-regulated or down-regulated compared to mock-treated controls (two-way ANOVA, FDR-corrected *P* ≤ 0.05). Treatment with Br-2′,3′-cAMP resulted in 1925 up-regulated and 1958 down-regulated genes at 30 min. This number further increased after 6 hours, with 2937 genes up-regulated and 2630 down-regulated, indicating a progressive transcriptional response over time. Similarly, Br-3′,5′-cAMP treatment led to 1249 up-regulated and 1312 down-regulated genes at 30 min, increasing to 2690 and 2429, respectively, by 6 hours (Fig. 5A). Despite comparable accumulation of both isomers after 30 min (Fig. 1C), 2′,3′-cAMP induced a more extensive and rapid transcriptional response, suggesting stronger early signaling activity.

The analysis of early DEGs (30 min) revealed a partially overlapping but largely isomer-specific set of gene expression changes (Fig. 5, B and C). Genes up-regulated by 2′,3′-cAMP were strongly enriched in stress-related processes, including the regulation of carbon utilization, response to putrescine, and spermine catabolism (Fig. 5D). Additional enrichment in oxylipin and jasmonic acid biosynthetic processes; photorespiration; and responses to hypoxia, ROS, wounding, water deprivation, salt stress, and bacterial infection suggest that 2′,3′-cAMP triggers a broad stress-adaptive transcriptional program, consistent with its proposed role in stress signaling (*14*). In contrast, genes up-regulated by 3′,5′-cAMP were enriched in a more limited set of processes, notably response to zinc ion starvation, cell wall modification, and cellular response to oxygen levels (Fig. 5D), reflecting a more focused role in nutrient sensing and structural adaptation.

Down-regulated genes also showed divergent functional signatures between the two treatments. Following 2′,3′-cAMP treatment, down-regulated genes were enriched in pathways related to protein N-linked glycosylation, base-excision repair, chaperone-mediated protein folding, and membrane protein localization (Fig. 5E), indicating the suppression of protein processing, DNA repair, and biosynthetic regulation. In contrast, 3′,5′-cAMP treatment predominantly affected genes involved in photosystem II repair, chloroplast organization, and gene expression, pointing to a more targeted repression of chloroplast maintenance and transcriptional activity.

Notably, a number of genes were commonly regulated by both treatments, suggesting a partially shared response. Shared up-regulated genes were enriched in extracellular transport, sterol transport, and general stress responses such as cellular response to hypoxia and cold (Fig. 5F), indicating convergence on environmental stress adaptation. On the other hand, shared down-regulated genes were associated with embryo sac egg cell differentiation, translational termination, chloroplast organization, ribonucleoside metabolism, and response to endoplasmic reticulum stress, reflecting a shared suppression of growth, organelle function, and biosynthetic capacity.

Together, these results demonstrate that 2′,3′-cAMP and 3′,5′-cAMP elicit distinct yet partially overlapping transcriptional programs. While 2′,3′-cAMP triggers a broad, stress-adaptive reprogramming of gene expression, 3′,5′-cAMP appears to fine-tune responses related to nutrient status and cellular homeostasis.

### Developmental responses to cAMP isomers in *Arabidopsis*

Next, we tested the effects of cAMP isomers on early seedling establishment by germinating *Arabidopsis* seeds on medium supplemented with Br-2′,3′-cAMP or Br-3′,5′-cAMP at concentrations of 0, 10, and 100 μM (fig. S4A). We observed that germination rates were comparable across all concentrations tested, indicating that neither cAMP isomer altered seed germination under these conditions.

We next examined cAMP isomers effect on postgermination development at 7 days after germination under the same conditions. Notably, 7-day-old seedlings grown on 2′,3′-cAMP at 10 and 100 μM displayed a significant increase in primary root length relative to control (fig. S4, B and C). In contrast, 3′,5′-cAMP showed no detectable effect at 10 μM, but promoted root elongation at 100 μM, implying a dose-dependent response that may reflect a higher activation threshold or lower signaling efficiency (fig. S4, B and C).

To determine whether cAMP also influences shoot growth, we quantified cotyledon and true leaf blade area in 15-day-old seedlings (fig. S4, D and E). Treatment with 2′,3′-cAMP produced only modest changes in leaf expansion, whereas 3′,5′-cAMP significantly increased cotyledon and leaf area, particularly at 100 μM (fig. S4, D and E), indicating a more specific effect of 3′,5′-cAMP on shoot development.

Overall, these phenotypic data support that 2′,3′-cAMP and 3′,5′-cAMP act as distinct but partially overlapping regulators of growth, with effects that are both dose and developmental stage dependent.

## DISCUSSION

Our comprehensive analysis of 2′,3′-cAMP and 3′,5′-cAMP in *Arabidopsis* reveals previously uncharacterized complexity in cAMP signaling in plants. The results demonstrate that these structural isomers are produced by distinct enzymatic mechanisms from distinct substrates and elicit partly overlapping yet largely distinct cellular responses, supporting their roles as discrete signaling molecules with specific targets and biological functions.

### Distinct enzymatic origins of 2′,3′-cAMP and 3′,5′-cAMP

One of the key findings of this study is that 2′,3′-cAMP and 3′,5′-cAMP are generated by different cAMP-producing enzymes with distinct substrate specificities. Plant ACs such as previously identified AFB5 (*8*) and HpAC1 (*21*) catalyze the formation of 3′,5′-cAMP from ATP but produce minimal 2′,3′-cAMP when incubated with nucleic acid substrates (Fig. 2, A to F). Similarly, a mammalian AC preferentially synthesizes 3′,5′-cAMP from ATP (Fig. 2, J to L), with catalytic activity significantly enhanced by FSK, a small molecule known to bind a hydrophobic pocket at the interface of the C1a and C2 domains, stabilizing the catalytically active conformation (*22*). In mammals, membrane-bound AC activity is tightly regulated by heterotrimeric G-proteins, primarily the Gαs subunit, and FSK further amplifies this activation (*24*).

Notably, in the absence of FSK stimulation, the reconstituted mammalian AC system (VC_1_ + IIC_2_) produced 3′,5′-cAMP at a rate comparable to AFB5 and L7^TIR^ (Fig. 2, C, I, and L), suggesting that the relatively moderate activity of plant ACs may reflect the absence of specialized activation factors, such as Gαs or small-molecule modulators. Given that plant ACs are structurally distinct and that plants lack known Gαs-equivalent signaling components, it is plausible that plant ACs are activated by alternative regulatory mechanisms, possibly linked to environmental stimuli, which requires further investigation.

In our experimental conditions, HpAC1 exhibited a limited AC activity compared to previous reports (*21*). Structural studies have shown that HpAC1 lacks essential cyclase residues in its active site and displays no measurable AC activity under crystallographic analysis (*25*). This structural deficiency likely accounts for the low activity observed in our assays.

The previously characterized L7^TIR^ RNA/DNA hydrolases (*9*) exhibited dual activity, producing both 2′,3′-cAMP from nucleic acids and 3′,5′-cAMP from ATP. While L7^TIR^ generated significant amounts of 2′,3′-cAMP, the rate of 3′,5′-cAMP production was comparatively higher (Fig. 2, G to I). This dual specificity is consistent with L7^TIR^ role in TIR-mediated immune responses in plants (*26*) and suggests that 2′,3′-cAMP may act as a fine-tuning element in immune signaling networks. The identification of ACs with dual or intermediate activities highlights an evolutionary diversification of cyclase functions and suggests a more intricate regulation of cAMP signaling than previously suspected.

These findings extend the traditional view that CNP signaling is mediated solely through the canonical 3′,5′-cyclization pathway. Instead, they support the emerging concept of an additional 2′,3′-cAMP–based pathway, which is implicated in plant immune responses and known to be essential for tissue protection during traumatic brain injury in mammals (*27*). The substrate and product specificities of enzymes that generate distinct cAMP isomers provide a mechanistic basis for the independent regulation of these cAMP pools: While 3′,5′-cAMP is generated from ATP by ACs under normal physiological conditions, 2′,3′-cAMP produced by RNA/DNA hydrolysis is likely coupled to RNA turnover or DNA metabolism. This dual-origin model explains how plants can maintain distinct pools of CNPs and selectively activate downstream pathways tailored to specific cellular needs, balancing growth, stress adaptation, and defense responses.

### Specific and partially shared signaling networks downstream of cAMP isomers

Our multiomics approach has revealed that 2′,3′-cAMP and 3′,5′-cAMP trigger partially overlapping but largely distinct cellular responses. The transcriptomic profiling demonstrates that both cAMP isomers induce widespread transcriptional reprogramming, with certain overlap between their responses and distinct, isomer-specific signatures (Fig. 5). Similarly, proteomic and metabolomic analysis show divergent patterns of protein abundance and metabolites changes, with each isomer preferentially affecting different biological processes (Figs. 3 and 4).

Particularly noteworthy is the apparent specialization of these signaling pathways: 2′,3′-cAMP predominantly influences RNA metabolism, translation, and nuclear processes, whereas 3′,5′-cAMP more strongly affects energy metabolism, photosynthesis, and stress responses. This functional divergence is consistent with their distinct biogenesis: 2′,3′-cAMP derives from RNA processing (*9*, *14*) and is therefore appropriately to regulate RNA-related functions, while 3′,5′-cAMP is generated from ATP and preferentially engages energy- and growth-related processes, which may be related to 3′,5′-cAMP involvement in auxin signaling (*8*, *15*).

Consistently, exogenous 3′,5′-cAMP modestly enhanced primary root growth in 7-day-old Col-0 seedlings (fig. S4, B and C) and promoted leaf expansion (fig. S4, D and E), which is in line with reported roles for cAMP in controlling primary and lateral root development (*28*). A possible mechanistic basis for this enhancement may be due to cAMP-dependent regulation of cation fluxes via CNP-gated channels (CNGCs), with downstream effect on Ca^2+^-regulated growth machinery and cell expansion (*12*). Likewise, *B. napus* AC overexpression lines exhibit widened leaf blades alongside increased root length and biomass, attributed to cAMP-associated Indole-3-Acetic Acid (IAA) hyperaccumulation in leaf primordia (*18*).

Notably, only 2′,3′-cAMP, but not 3′,5′-cAMP, has been shown to induce SG formation (*13*, *29*). SGs are cytosolic condensates that form in response to stress and contain mRNAs, proteins, and metabolites (*30*, *31*). The ability of 2′,3′-cAMP to selectively trigger SG formation may lead to spatial and temporal redistribution of cellular components, potentially explaining its distinct impact on cellular homeostasis compared to 3′,5′-cAMP.

For the 2′,3′-cAMP treatment, enhanced primary root growth was also observed in 7-day-old seedlings (fig. S4, B and C). This response may reflect a priming-like mechanism in which plants pre-emptively reprogram cellular pathways to improve plant resilience to stress conditions. Previous studies have reported that exogenous 2′,3′-cAMP elicit a broad range of stress responsive changes, including accumulation of glucosinolates, protein/RNA degradation intermediates, and induction of molecular chaperones (*14*). Notably, these responses include increased accumulation of developmental regulators such as auxin-responsive factor 11 (ARF11), which modulates cell elongation and the transition toward differentiation during root development (*32*). However, whether this 2′,3′-cAMP–induced priming directly underlies the early root elongation phenotype observe here awaits further investigation.

Nevertheless, the observed overlap in regulated genes and proteins suggests a degree of convergence in downstream signaling, potentially through shared effector proteins. This cross-talk may allow for integration and fine-tuning of cellular responses to complex environmental stimuli. The temporal dynamics of these responses, with rapid changes in some pathways and delayed effects in others, further suggest a cascade-like propagation of signaling events following cAMP perception.

In addition, some overlap may happen due to the nonspecific cross-binding of the isomers to the cAMP target proteins. Given that the specificity of cAMP action is at least partly due to the spatial, subcellular compartmentalization of the cAMP isomers (*15*, *33*), our exogenous treatments may likely overcome those regulations.

### Evolutionary implications and biological significance

The coexistence of two parallel cAMP signaling systems in plants raises intriguing questions regarding their evolutionary origins and biological significance. The predominance of 2′,3′-cAMP production and metabolic regulation as compared to 3′,5′-cAMP in plants (Fig. 1D) contrasts with the strong preference for 3′,5′-cAMP synthesis in mammalian system. This suggests an evolutionary divergence of cAMP signaling mechanisms across kingdoms that may reflect adaptation to distinct environmental challenges or differences in cellular organization between plants and animals.

From a functional perspective, maintaining distinct yet interconnected cAMP pathways offer several potential advantages. First, it enables fine-tune regulation of cellular processes, with each isomer selectively controlling specific aspects of metabolism, gene expression, and stress responses. Second, it allows cyclase activity to be differentially regulated through distinct stimuli—ATP levels driving 3′,5′-cAMP production versus nucleic acid turnover regulating 2′,3′-cAMP synthesis—providing multiple layers of responsiveness to cellular status. Third, the presence of two cAMP pathways may not only provide ppotential cross-talk platform but also confer both redundancy and specialization, enhancing the robustness and versatility of plant adaptive responses.

Our GO enrichment analyses further support this model, indicating that 2′,3′-cAMP is closely associated with stress-responsive pathways linked to RNA metabolism, including potential regulation of SG dynamics or alternative splicing under adverse conditions (Figs. 4 and 5). In contrast, 3′,5′-cAMP appears more prominently involved in regulating core metabolic pathways and maintaining energy homeostasis. This functional specialization likely enables plants to orchestrate complex physiological responses to environmental stresses through parallel yet distinct signaling networks.

Together, our findings provide a framework for understanding cAMP signaling complexity in plants, highlighting evolutionary innovation and functional diversification. Future work dissecting the precise molecular targets, regulatory mechanisms, and evolutionary conservation of these pathways will be crucial to fully unraveling their biological significance.

### Methodological limitations

Our use of brominated analogs provides a powerful tool to track cAMP metabolism and biological effects in plants; however, this approach also introduces potential limitations. Although Br-2′,3′-cAMP and Br-3′,5′-cAMP closely mimic their natural counterparts, their cellular uptake, metabolic stability, or binding affinities may differ slightly from those of endogenous molecules. Moreover, the shared products of Br-2′,3′-cAMP and Br-3′,5′-cAMP degradation may further confound the results. Therefore, future studies using genetic approaches to selectively manipulate endogenous cyclase activities will be critical to complement and validate the pharmacological findings reported here.

Several important questions emerge from our study. First, what are the specific targets or binding proteins for 2′,3′-cAMP apart from identified RBP47b (*13*), and how do they differ from the well-characterized 3′,5′-cAMP targets from animals such as CNGCs (*34*) and cAMP-dependent protein kinases (PKAs) (*35*). Identifying these 2′,3′-cAMP binding proteins will be essential to understanding the unique signaling pathways it governs. Second, how ACs differentially regulated in response to environmental stimuli or developmental cues in plants in the absence of classical Gαs-mediated activation mechanisms. Last but not least, to what extent do 2′,3′-cAMP and 3′,5′-cAMP signaling systems interact—either synergistically or antagonistically—under specific physiological conditions. Addressing these questions will require further biochemical characterization of CNP-binding proteins and detailed investigation of cyclase regulation across various stress and developmental contexts.

In addition, comparative analyses across a broader range of plant species will be important to determine the evolutionary conservation of these dual cAMP signaling systems. Broader taxonomic sampling will be necessary to establish the phylogenetic distribution, functional divergence, and potential specialization of these pathways across the plant kingdom.

In conclusion, our study provides compelling evidence that 2′,3′-cAMP and 3′,5′-cAMP represent distinct yet interconnected signaling systems in plants, arising from different enzymatic pathways and regulating overlapping but specialized sets of cellular processes. The preferential production and metabolic control of 2′,3′-cAMP in plants challenges the traditional focus on 3′,5′-cAMP as the predominant CNP intracellular messenger and opens avenues for understanding the complexity of plant signaling networks.

By establishing the distinct origins and effects of these cAMP isomers, our work provides a foundation for future studies aimed at elucidating their specific roles in plant development, stress responses, and adaptation to environmental challenges. Understanding these parallel signaling pathways may ultimately inform strategies for enhancing crop resilience and productivity in changing environmental conditions.

## MATERIALS AND METHODS

### 2′,3′-cAMP and 3′,5′-cAMP isolation from plant samples

Lyophilized material was homogenized using a ball mill, and 10 mg of the sample was extracted with 600 μl of 4% acetic acid, 10 μl of 100 mM IBMX (in ethanol), and 5 μl of an isotopically labeled internal standard of 3′,5′-^13^C5-cAMP (1 × 10^−7^ mol·liter^−1^, LGC Standards, France, www.lgcstandards.com/FR/en/p/TRC-A280457). The mixture was vortexed for 30 s and centrifuged at 10,000*g* at 4°C for 5 min. The supernatant was transferred to a precooled 2-ml Eppendorf tube, mixed with 1200 μl of acetonitrile, vortexed again for 30 s, and centrifuged under the same conditions. The resulting supernatant was transferred to a 15-ml Falcon tube and combined with 200 μl of MS-grade water, 200 μl of 100 mM ammonium formate/acetonitrile (5:95, v/v), and 2000 μl of acetonitrile. After vortexing for 1 min, the mixture was centrifuged for 5 min. Solid-phase extraction (SPE) was performed using HyperSep Si (500 mg; Thermo Fisher Scientific, USA) columns preconditioned with 5 ml of MS water and equilibrated with 2.5 ml of 100 mM ammonium formate/acetonitrile (5:95, v/v). Samples were slowly loaded onto the columns, followed by a wash with 1 ml of the equilibration buffer. Analytes were eluted with 2 ml of MS water into clean 2-ml Eppendorf tubes, frozen at −80°C, lyophilized, and stored at −80°C until LC-MS analysis. Before analysis, samples were reconstituted in 40 μl of MS water, centrifuged, and transferred to LC vials. Injected volume was 10 μl.

### LC-MS analysis of 2′,3′-cAMP and 3′,5′-cAMP

The quantitative analysis of 2′,3′-cAMP and 3′,5′-cAMP was performed using an LC-MS system comprising an ACQUITY UPLC I-Class coupled to a Xevo TQ-S triple quadrupole mass spectrometer (Waters, Milford, MA, USA) equipped with an electrospray ionization (ESI) source. Chromatographic separation was achieved on an ACQUITY UPLC HSS T3 column (1.8 μm, 2.1 mm by 100 mm; Waters, USA) using a binary mobile phase system consisting of acetonitrile (phase A) and 15 mM ammonium formate at pH 3.96 (phase B). The column was maintained at 50°C with a constant flow rate of 0.4 ml/min. The gradient program was as follows: 0% A from 0 to 2 min, linear increase to 12% A over 10 min, followed by an increase to 15% A over 1 min, and re-equilibration at 0% A for 5 min.

### Recombinant protein expression and purification

MBP-fused L7^TIR^ (*9*) construct and glutathione *S*-transferase (GST)–HpAC1 (*21*) were described previously. The coding sequences of AFB5 (AT5G49980) from *Arabidopsis* and mammalian AC fragments IIC2 and VC1 were cloned into the pMAL-c6T vector using primers listed in table S1. The resulting plasmids were introduced into BL21 competent cells (NEB, C2530H) to produce MBP-fusion proteins with additional N-terminal 6 × His tags. The transformants were grown in LB medium containing ampicillin (100 μg ml^−1^) and 2% glucose at 37°C. Fusion protein expression was induced by adding isopropyl-β-d-thiogalactopyranoside to a final concentration of 0.5 mM at *A*_600_ of 0.6, followed by incubation at 16°C for 12 hours. Cells were collected by centrifugation, resuspended in lysis buffer [25 mM tris-HCl (pH 8.0), 150 mM NaCl, 5 mM EDTA, 5 mM EGTA, 1% (v/v) Triton X-100, 1 mM phenylmethylsulfonyl fluoride, lysozyme (0.1 mg ml^−1^)] and lysed by sonication. The cell lysate was clarified by centrifugation at 18,000*g* for 35 min, and the supernatant was applied to amylose resin (NEB, catalog no. E8021L) or glutathione agarose resin (Thermo Fisher Scientific, catalog no. 16102BID). After extensive washing with five column volumes of buffer containing 50 mM tris-HCl (pH 8.0) and 150 mM NaCl, bound proteins were eluted with 10 mM maltose (amylose resin) or 10 mM glutathione (glutathione resin) in 50 mM tris-HCl (pH 8.0). For AFB5, the eluted fusion protein was digested with Tobacco Etch Virus (TEV) protease at 4°C overnight to remove the N-terminal MBP-His_6_ tag. The cleaved protein was concentrated and further purified by size exclusion chromatography using the Superdex 75 Increase 10/300 column (Cytiva) for subsequent enzymatic kinetics measurements. L7^TIR^ was purified using the same affinity and size exclusion workflow. However, the removal of the N-terminal MBP-His tag using PreScission protease caused substantial aggregation, which is consistent with previous reports showing that L7^TIR^ forms large aggregates and filamentous assemblies composed of dsDNA/RNA binding tetramers after MBP cleavage (*9*). Therefore, the MBP-L7^TIR^ fusion protein was used for enzymatic kinetics assays, as it remained soluble and active after size exclusion chromatography. The homogeneity and purity of eluted protein fraction was analyzed by 10% SDS–polyacrylamide gel electrophoresis with Coomassie blue gel staining.

### Production and detection of 2′,3′-cAMP and 3′,5′-cAMP in vitro

For 3′,5′-cAMP production, the reaction mixture consisted of 10 mM tris-HCl (pH 8.0), 1 mM MgCl_2_, 1 mM MnCl_2_, 1 mM ATP, 1 mM dithiothreitol (DTT), and 5 μg of recombinant protein in a final volume of 100 μl. To assess the effect of FSK on mammalian ACs, 10 μM FSK was added to the reaction mixture. Reactions were incubated at 30°C for 25 min. Enzyme activity was terminated by heating the samples at 100°C for 10 min, followed by centrifugation at 12,000*g* for 10 min. For 2′,3′-cAMP production, recombinant proteins were incubated with 1 μg of *Arabidopsis* genomic DNA or total RNA in a buffer containing 25 mM tris-HCl (pH 8.0) and 150 mM NaCl, in a total volume of 100 μl. Reactions were carried out at 25°C for 16 hours and centrifuged at 12,000*g* for 10 min. The resulting supernatant was subjected to LC-MS/MS analysis for metabolite identification and quantification. The cAMP isomers quantification was normalized against the corresponding standard curve.

In vitro reaction samples were filtered through precleaned 30,000 Molecular weight cut off (MWCO) HY tube filter (Vivacon 500, Sartorius, UK) at 12,000 rcf for 30 min. The filter was precleaned with water (LC/MS grade, HiPerSolv CHROMANORM, VWR Chemicals, France). Samples were frozen at −20°C before LC/MS analysis. LC/MS analysis was performed on Ultimate 3000 UHPLC chromatograph (Thermo Fisher Scientific, US) connected to Q Exactive HF mass spectrometer (Thermo Fisher Scientific, US). cAMP was separated from the matrix components on ACQUITY UPLC HSS T3 C18 column (1.8 μm, 1.0 mm by 100 mm; Waters, US) connected in-line with HSS T3 precolumn (1.8 μm, 2.1 mm by 5 mm; Waters, US) maintained at 30°C with 8 min. Separation was achieved over a gradient elution from 1 to 10% of mobile phase B, where mobile phase A was 0.1% formic acid (98 to 100% for HPLC, LiChropur, Supelco, Germany) in water (LC-MS grade, HiPerSolv CHROMANORM, VWR Chemicals, France) and mobile phase B was 80% acetonitrile (LC/MS grade, OPTIMA, Thermo Fisher Scientific, UK) in water (LC/MS grade, HiPerSolv CHROMANORM, VWR Chemicals, France) containing 0.1% formic acid (98 to 100% for HPLC, LiChropur, Supelco, Germany). Elution was followed by 2 min of washing the column with 75% B and 10 min of re-equilibration with 1% B. The LC flow rate was set to 80 μl/min. Injection volume was 10 μl for the standard compounds and tested in vitro reactions.

Q Exactive HF was equipped with HESI ion source and CID fragmentation cell. Source parameters were set to: spray voltage of 3000 V, capillary temperature of 250°C, sheath gas of 33, aux gas of 10, and S-lens RF level of 50 V. Data used for quantification were acquired in the negative-ion mode, and the parallel reaction monitoring (PRM) transition of 328.0447 to 134.0468 was monitored for cAMP detection (both isomers). Full scan data were acquired at 60,000 resolution, PRM scan data at 30,000 with isolation window set to 3 mass-charge ratio (*m/z*), collision energy set to 40 (NCE), automatic gain control target of 1 × 10^6^, and maximum and IT set to 100 ms. Quantification was performed using external calibration method with minimum six calibration points using 2′,3′-cAMP (≥93%, monophosphate sodium salt, Sigma-Aldrich, US) and 3′,5′-cAMP (≥98.0% HPLC, monophosphate sodium salt monohydrate, Sigma-Aldrich, US) as analytical standards.

### Plant growth conditions and cNMP treatment

*Arabidopsis thaliana* Col-0 seedlings were grown in liquid Murashige and Skoog medium (*36*) supplemented with 1% (v/v) sucrose under continuous light conditions. The medium was replaced after 7 days, and on day 10, treatments with 1 μM Br-2′,3′-cAMP (catalog no. B280, Biolog Life Science Institute, Bremen, Germany) and 1 μM Br-3′,5′-cAMP (catalog no. B5386, Sigma-Aldrich, St. Louis, MO, USA) were initiated. The 1 μM concentration was chosen on the basis of previously reported low- to mid-micromolar levels of endogenous 2′,3′-cAMP in *Arabidopsis* lysates (*13*). Water, used as the solvent for compound dissolution, served as a treatment control. Samples were collected at 15 min and 30 min, and at 1 hour, 6 hours, and 24 hours posttreatment. After gently blotting on paper towels, seedlings were flash-frozen in liquid nitrogen. The frozen material was vacuum-dried for 3 days before further analysis.

For developmental phenotype measurement, seeds were surface sterilized with 50% bleach followed by a wash with 70% ethanol containing 0.05% Tween 20 and then washed three times with sterile distilled water. After stratification at 4°C in darkness for 2 days, the sterilized seeds were sown on solid 0.5× Murashige and Skoog medium (pH 5.7) containing Br-2′,3′-cAMP or Br-3′,5′-cAMP at indicated concentration. Seedlings were grown in square petri dishes in a vertical position inside a Percival CU-41 L5 growth chamber under long-day photoperiod conditions (16-hour light/8-hour dark) at 22° ± 2°C with a light intensity of 120 μmol m^−2^ s^−1^.

### Developmental phenotype analysis

The germination rate of *Arabidopsis* Col-0 was evaluated under control and treatment conditions. Seeds were scored every 24 hours for 5 days, with germination defined by visible radicle emergence. Three biological replicates were analyzed with 35 seeds per replicate, and the germination rate was calculated as the cumulative percentage of germinated seeds relative to the total number of seeds sown per plate.

For primary root measurements, seedlings were grown vertically on 0.5× Murashige and Skoog medium with or without Br-2′,3′-cAMP or Br-3′,5′-cAMP (10 or 100 μM). Plates were imaged at 7 days after germination, and primary root length was measured from the hypocotyl-root junction to the root tip using Fiji (*37*). Measurements were collected from three biological replicates (*n* = 15 roots).

For heteroblastic analysis, seedlings were grown for 15 days on 0.5× Murashige and Skoog medium with or without Br-2′,3′-cAMP or Br-3′,5′-cAMP (10 or 100 μM). Cotyledons and successive true leaves were excised, placed on fresh plates, and arranged by developmental stage from cotyledons to true leaves. Plates were imaged, and cotyledons and leaf area were quantified using Fiji (*37*). Area measurements were obtained from three biological replicates (*n* = 15 leaves).

### Metabolite and protein extraction

Metabolite and protein extraction was carried out using a modified protocol previously described (*38*). Briefly, 10 mg of vacuum-dried plant tissue powder was subjected to a biphasic extraction using a methyl tert-butyl ether (MTBE)/methanol/water solvent system. This process partitioned the sample into three distinct phases: an aqueous phase enriched in primary and secondary metabolites, an organic phase containing lipids, and a protein-rich pellet. Equal volumes of each phase were dried using a centrifugal evaporator and stored at −80°C for downstream metabolomics and proteomics analyses.

### LC-MS metabolomics for secondary metabolite identification

The dried aqueous phase was analyzed via ultra-performance LC (UPLC) coupled to an Exactive Orbitrap mass spectrometer (Thermo Fisher Scientific, Bremen, Germany), operating in both positive and negative electrospray ionization modes. The method was adapted from previously described protocol (*39*), with full-scan mode acquisition. Raw data were processed using REFINER MS version 10.5 (GeneData; www.genedata.com), involving peak detection, chemical noise removal, retention time (RT) alignment, and isotopic peak clustering.

Metabolite features were annotated by comparison to an in-house reference library of authentic standards using *m*/*z* deviations of ≤10 parts per million and RT differences of ≤0.1 min. Chromatographic data from standards were used to define predominant adducts and RTs. In addition, phenylpropanoids, flavonols, and glucosinolates were identified on the basis of characteristic fragmentation patterns and elemental composition using the same instrumentation and settings as the method previously described (*40*).

### LC-MS/MS for protein analysis and data processing

Protein pellets from the MTBE extraction were solubilized in 100 μl of urea-thiourea buffer (6 M urea and 2 M thiourea in 40 mM ammonium bicarbonate). Protein concentration was determined using the Bradford assay (Carl Roth, Karlsruhe, Germany). A total of 40 μg of protein was reduced with 5 mM DTT for 30 min at room temperature, followed by alkylation with 15 mM iodoacetamide for 20 min in the dark. Proteins were enzymatically digested using a LysC/trypsin mix (Promega, Fitchburg, WI, USA) according to the manufacturer’s instructions. After digestion, samples were acidified with trifluoroacetic acid (TFA) to pH < 2, desalted using C18 Empore extraction discs (3M, Maplewood, MN, USA) and STAGE tips (*41*), and concentrated to ~4 μl by centrifugal evaporation. Peptides were stored at −80°C until LC-MS/MS analysis.

For LC-MS/MS, peptides were reconstituted in loading buffer (2% acetonitrile and 0.2% TFA), and 0.8 to 1.0 μg were analyzed using a reverse-phase column on a Q Exactive Plus or Q Exactive HF mass spectrometer (Thermo Fisher Scientific, Waltham, MA, USA). Raw files were processed with MaxQuant version 1.6.0.16 (*42*) using the Andromeda search engine (*43*). Protein identification was performed against the *Arabidopsis* TAIR10 reference proteome (updated December 2017), along with a contaminant database included in MaxQuant. Hits identified as contaminants or decoys were removed from further analysis.

MaxQuant search parameters included trypsin and LysC as proteases, with up to two missed cleavages allowed. The carbamidomethylation of cysteine was set as a fixed modification, while methionine oxidation was included as a variable modification. The FDR was controlled at <1% at the protein level. Label-free quantification (LFQ) and “match between runs” options were enabled. Only proteins identified by at least one unique and one razor peptide were quantified, and a minimum peptide length of six amino acids was required. For downstream analyses, LFQ intensities were used, and protein groups were retained only if supported by at least two unique peptides. Common contaminants such as keratins were excluded from the dataset.

### Statistical analysis

Both metabolite and protein datasets were log_2_-transformed before two-way ANOVA, performed in MeV (*44*) with treatment (treated versus untreated) and time (15 min, 30 min, 1 hour, 6 hours, and 24 hours) as factors. Resulting *P* values were adjusted using FDR correction to identify metabolites and proteins significantly affected by treatment.

For germination rate and primary root length, statistical significance was assessed using one-way ANOVA followed by Tukey’s multiple comparison test. Cotyledon and leaf area data were analyzed using two-way ANOVA with Tukey’s multiple comparison test. Significance was indicated as *P < 0.05, **P < 0.01, and ***P < 0.001. Analyses were performed in GraphPad Prism version 10.1 (2019, GraphPad Software, San Diego, CA, USA). All the data analyzed were obtained from three independent experiments and presented as mean values (± SD).

### RNA extraction and RNA-seq analysis

A 10 mg of lyophilized tissue was used to extract total RNA using an RNA extraction kit (Macherey-Nagel). Its quality was assessed using a Bioanalyzer RNA 6000 Nano (Agilent, Santa Clara, CA, USA). RNA-seq was performed by Lexogen GmbH (Vienna, Austria) using QuantSeq 3′-mRNA library preparation and QuantSeq 3′-UTR NextSeq SR75 sequencing. Integrated RNA-seq data analysis was performed by Lexogen using the STAR aligner to map sequencing reads to the *A. thaliana* reference genome (Araport11). Gene-level read counts were quantified, and the differential expression analysis was conducted using the DESeq2 package in R (*45*). Obtained *P* values were adjusted using FDR correction to control multiple testing. The analysis included two biological replicates per treatment, each representing an independently treated seedling flask, at two points: 30 min and 6 hours posttreatment.
