# Supplementary material for: Biogenesis and downstream effects of 3′,5′ and 2′,3′ cAMP isomers in plants
Source: Sci Adv. 2026 May 8;12(19):eaea7828. doi: 10.1126/sciadv.aea7828 (PMC13155313; doi:10.1126/sciadv.aea7828)
Supplement: Supplementary file 1 — Figs. S1 to S4 Table S1 Legends for data S1 to S8 [file sciadv.aea7828_sm.pdf]

Science Advances
AAAS

Supplementary Materials for

Biogenesis and downstream effects of 3′,5′ and 2′,3′ cAMP isomers in plants

Mingyue Li *et al.*

Corresponding author: Monika Chodasiewicz, monika.chodasiewicz@kaust.edu.sa;
Aleksandra Skirycz, skirycza@msu.edu; Jiří Friml, jiri.friml@ist.ac.at

Sci. Adv. **12**, eaea7828 (2026)
DOI: 10.1126/sciadv.aea7828

The PDF file includes:

Figs. S1 to S4
Table S1
Legends for data S1 to S8

Other Supplementary Material for this manuscript includes the following:

Data S1 to S8

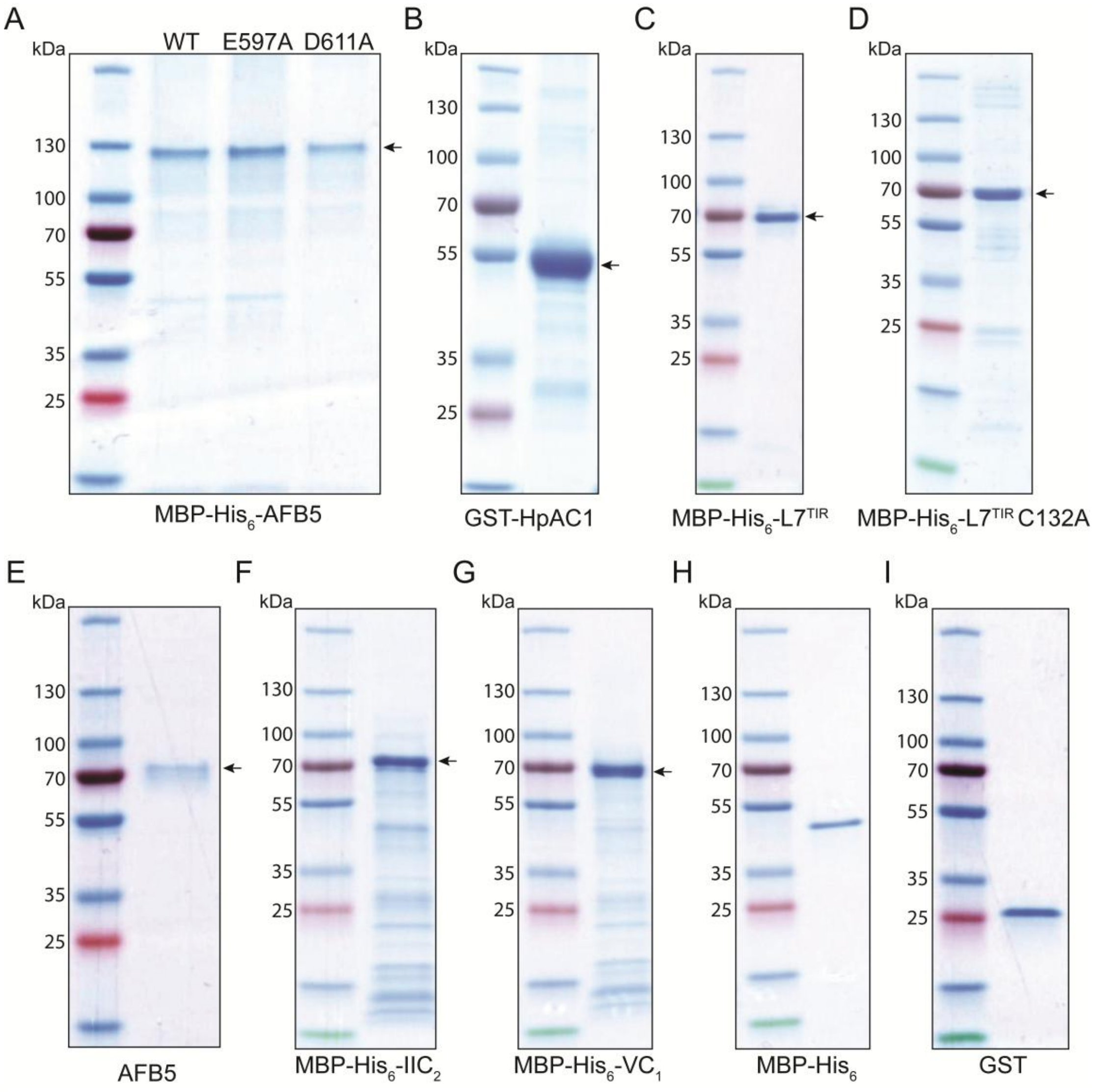

Fig. S1.

SDS-PAGE analysis confirming the purity of recombinant proteins used in this study. (A) MBP-His$_6$-AFB5: WT, E597A and D611A. **(B)** GST-HpAC1. **(C)** MBP-His$_6$-L7^{TIR}. **(D)** MBP-His$_6$-L7^{TIR} C132A. **(E)** AFB5 after cleavage of MBP tag. **(F)** MBP-His$_6$-IIC$_2$. **(G)** MBP-His$_6$-VC$_1$. **(H)** Free MBP-His$_6$ tag. **(I)** Free GST tag. All recombinant proteins were expressed in and purified from *E. coli* BL21 (DE3) cells. Samples were resolved by SDS-PAGE and stained with Coomassie Brilliant Blue. Experiments were repeated independently twice with consistent results.

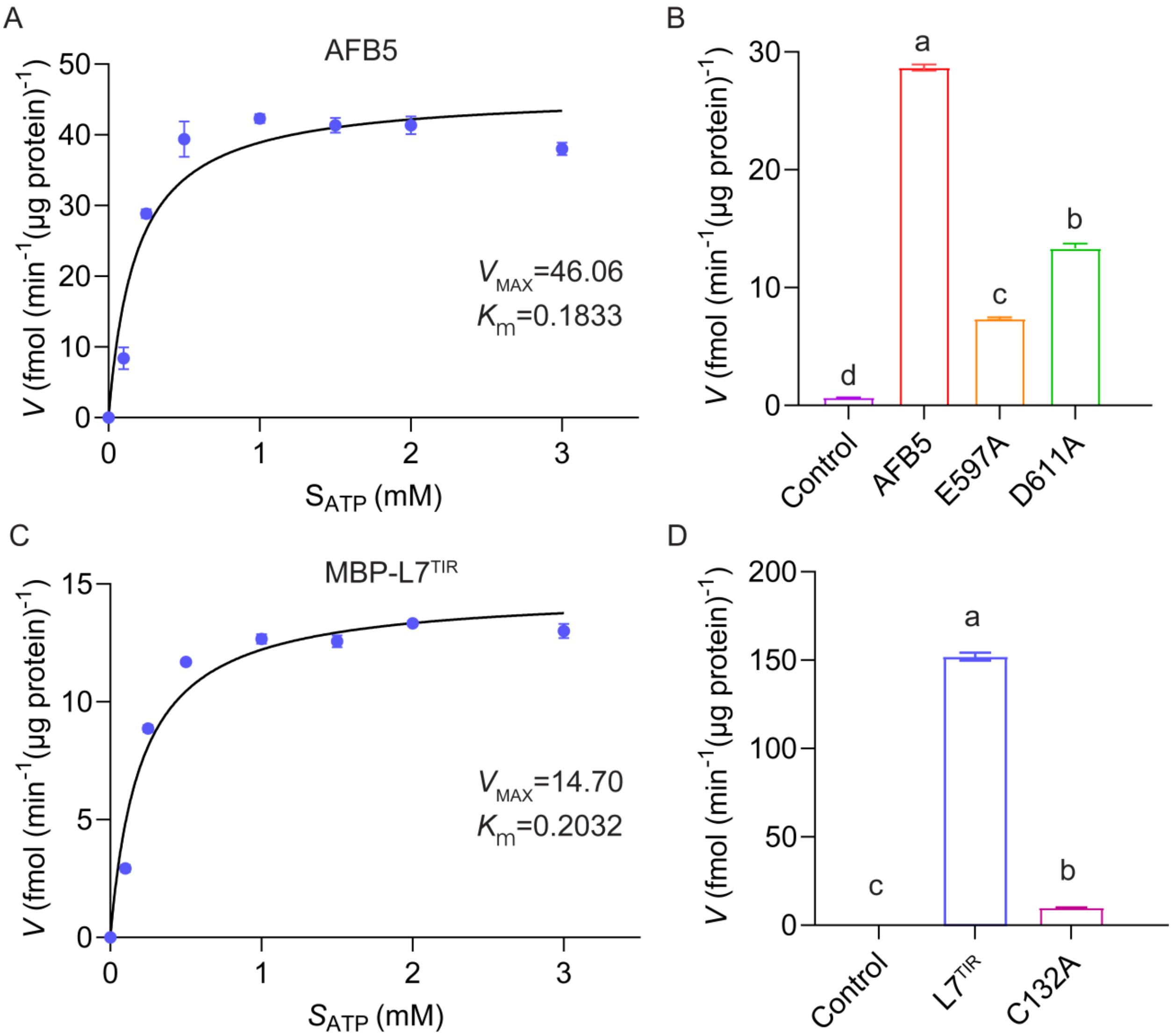

Fig. S2.

Michaelis–Menten kinetics and substitutional analysis confirming the AC or cyclic nucleotide synthesis activity of selected enzymes. (A) Michaelis–Menten kinetics for AFB5 *in vitro* AC activity quantified by LC–MS/MS. S_{ATP}, substrate (ATP) concentration; *V*, velocity. **(B)** *in vitro* AC activity assay of MBP-AFB5 and mutated variants E597A and D611A. **(C)** Michaelis–Menten kinetics for MBP-$L7^{TIR}$ AC activity. **(D)** *In vitro* 2′,3′-cAMP synthesis activity assay of $L7^{TIR}$ and mutant C132A. Data are shown as mean ± SD of three biological replicates.

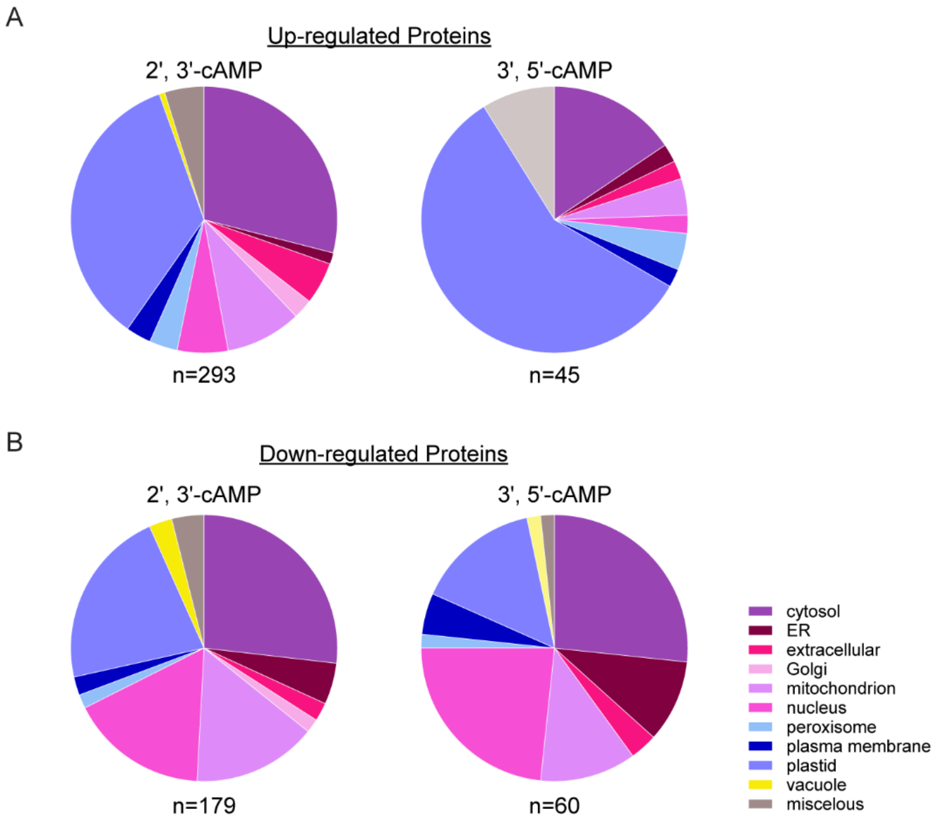

Fig. S3.

Subcellular distribution of significantly upregulated (A) and downregulated (B) proteins after Br-2′,3′-cAMP treatment. Subcellular localizations for each protein were identified using the SUBA5 database.

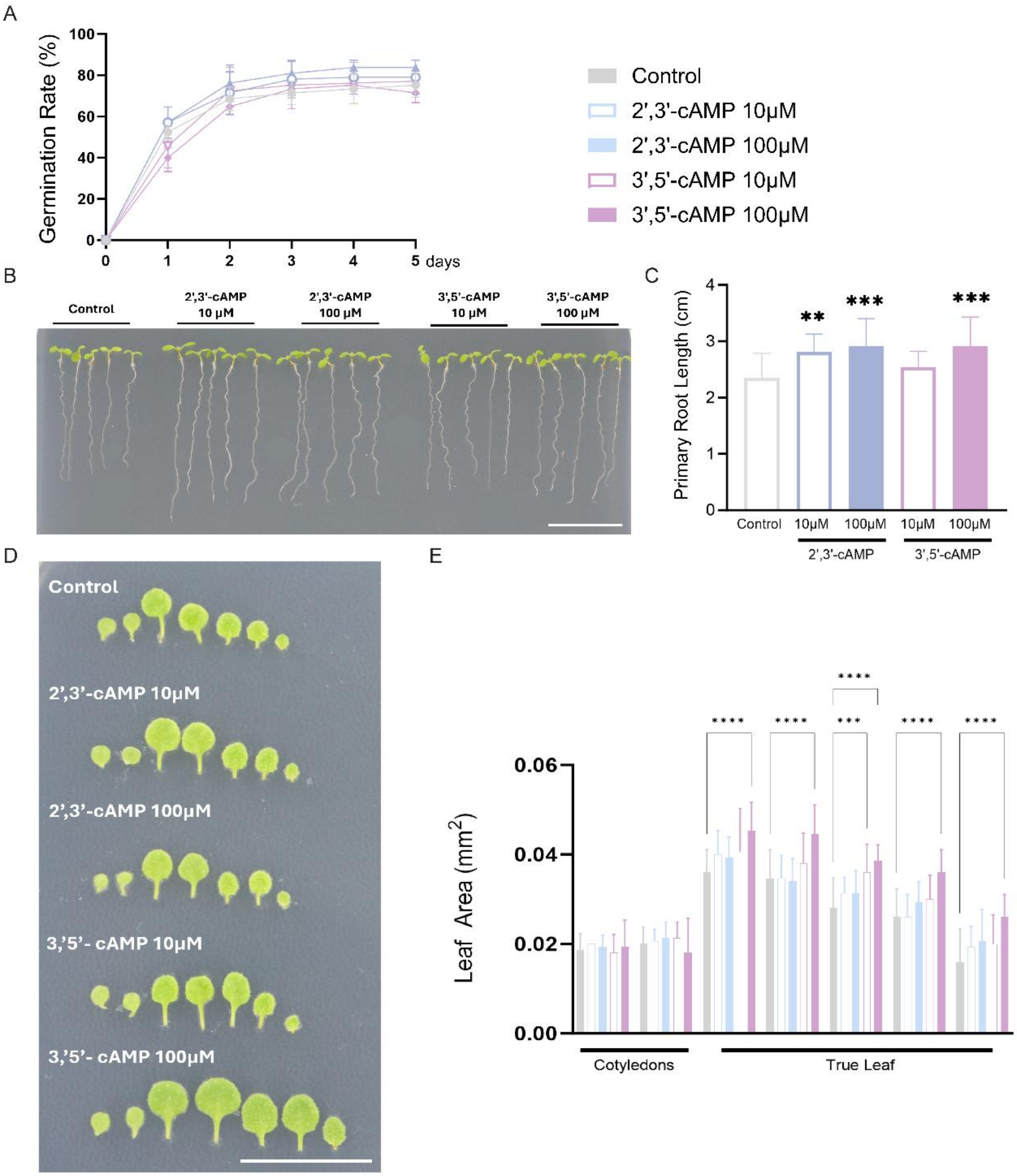

Fig. S4.

Effects of 2′,3′-cAMP and 3′,5′-cAMP on early Arabidopsis development. (A) Germination kinetics on medium supplemented with Br-2′,3′-cAMP (10 and 100 μM) or Br-3′,5′-cAMP (10 and 100 μM). Germination rate (%) was calculated as cumulative germinated seeds relative to the total number and plotted over five days. **(B)** Representative images of 7-day-old seedlings showing primary root length under control and cAMP treatments. **(C)** Quantification of primary root length in (B). n=15 seedlings. **(D)** Representative images of leaf heteroblastic assay. **(E)** Cotyledon and leaf blade area quantification across leaf positions shown in (F). Horizontal bars indicate mean ± SD (n = 15). Statistical significance is indicated as *$p < 0.05$, ** $p < 0.01$, *** $p < 0.001$. Scale bar = 1 cm.

Table S1.
Primers used in this study.

**Name**	**Sequence (5'-3')**
MBP-His_6-AFB5	
pMAL-c6t-AFB5-F	ACTTCCAGATGCTGATGGGCATGACACAAGATCGCTCAG
pMAL-c6t-AFB5-R	GGATCCGTCGACGATATCGCCTATAAAATCGTGACGAACTTTG
pMAL-c6t-ADCY2-F	GATGGGCGGCCGCGATAGACAGAGTGAATATTACTGTAGGTT
pMAL-c6t-ADCY2-R	CGGATCCGTCGACGATGGAAGCCAAGTTGCTCTGAGAA
pMAL-c6t-ADCY5-F	GATGGGCGGCCGCGATATGGAGATGAAAGCAGACATCAACG
pMAL-c6t ADCY5-R	CGGATCCGTCGACGATGTGTCCGATGGAGTTGGTTCTC
Sequencing	
Seq-F	GGTCGTCAGACTGTCGATGAAGCC
Seq-R	TGTCCTACTCAGGAGAGCGTTCAC

Data S1. (separate file)

Protein and metabolite analysis in response to Br-2′,3′-cAMP and Br-3′,5′-cAMP treatment.

Data S2. (separate file)

All genes annotated in RNAseq experiment after 30 min and 6 hr of treatment with Br-2′,3′-cAMP and Br-3′,5′-cAMP.

Data S3. (separate file)

Number of all DEGs after 30 min and 6 hr of treatment with Br-2′,3′-cAMP and Br-3′,5′-cAMP.

Data S4. (separate file)

Panther overrepresentation test for up-regulated genes specific for 30 min treatment with Br-2′,3′-cAMP and Br-3′,5′-cAMP, based on Fisher test.

Data S5. (separate file)

Panther overrepresentation test for down-regulated genes specific for 30 min treatment with Br-2′,3′-cAMP and Br-3′,5′-cAMP, based on Fisher test.

Data S6. (separate file)

Panther overrepresentation analysis of genes commonly regulated by both Br-2',3'-cAMP and Br-3′,5′-cAMP after 30min treatment, based on Fisher test.

Data S7. (separate file)

Panther overrepresentation analysis of proteins specifically up-regulated by Br-2',3'-cAMP or Br-3′,5′-cAMP treatment, based on Fisher test.

Data S8. (separate file)

Panther overrepresentation analysis of proteins specifically down-regulated by Br-2',3'-cAMP or Br- 3′,5′-cAMP treatment, based on Fisher test.
